# Biomedical Applications of Iron Oxide Nanoparticles: Current Insights Progress and Perspectives

**DOI:** 10.3390/pharmaceutics14010204

**Published:** 2022-01-16

**Authors:** María Gabriela Montiel Schneider, María Julia Martín, Jessica Otarola, Ekaterina Vakarelska, Vasil Simeonov, Verónica Lassalle, Miroslava Nedyalkova

**Affiliations:** 1 INQUISUR, Departamento de Química, Universidad Nacional del Sur (UNS)-CONICET, Bahía Blanca 8000, Argentina; gabriela.montielsc@gmail.com (M.G.M.S.); ma.julia.martin@gmail.com (M.J.M.); otarola_jessica@hotmail.com (J.O.); veronica.lassalle@uns.edu.ar (V.L.); 2 Faculty of Chemistry and Pharmacy, University of Sofia, 1 James Bourchier Blvd., 1164 Sofia, Bulgaria; e.vakarelska@gmail.com

**Keywords:** magnetite, SPION_S_, theranostics, hyperthermia, MRI, multitherapy, cancer, multimodal

## Abstract

The enormous development of nanomaterials technology and the immediate response of many areas of science, research, and practice to their possible application has led to the publication of thousands of scientific papers, books, and reports. This vast amount of information requires careful classification and order, especially for specifically targeted practical needs. Therefore, the present review aims to summarize to some extent the role of iron oxide nanoparticles in biomedical research. Summarizing the fundamental properties of the magnetic iron oxide nanoparticles, the review’s next focus was to classify research studies related to applying these particles for cancer diagnostics and therapy (similar to photothermal therapy, hyperthermia), in nano theranostics, multimodal therapy. Special attention is paid to research studies dealing with the opportunities of combining different nanomaterials to achieve optimal systems for biomedical application. In this regard, original data about the synthesis and characterization of nanolipidic magnetic hybrid systems are included as an example. The last section of the review is dedicated to the capacities of magnetite-based magnetic nanoparticles for the management of oncological diseases.

## 1. Introduction

### 1.1. General Concepts of Magnetic Nanotechnology

Nanotechnology is an interdisciplinary area between biology, chemistry, physics, materials science, biochemistry and engineering [1]. Although the concept of nanotechnology arose around 1960 with the emblematic conference of Richard Feynman, in which he raised the possibility of manipulating matter on an atomic and molecular scale [2], the term was popularized in the scientific area by Professor Norio Taniguchi from the Tokyo University of Sciences, who defined it as: “the treatment, separation, consolidation and deformation of materials into atoms or molecules” [3]. The basic idea of this definition was extended and explored in greater depth throughout the 1980s, driven by the development of experimental techniques that allowed the analysis of structures at the nano-scale, specifically from the emergence of atomic force microscopy (AFM) and tunnel effect (STM). This is how, from the discovery of quantum dots in 1981, followed by the discovery of fullerenes in 1985 and the publication in 1986 of K. Eric Drexler’s book: Engines of Creation: The Coming Era of Nanotechnology, began intensive research based on nanotechnology, which has led to great advances and new paradigms.

**Figure 1:**
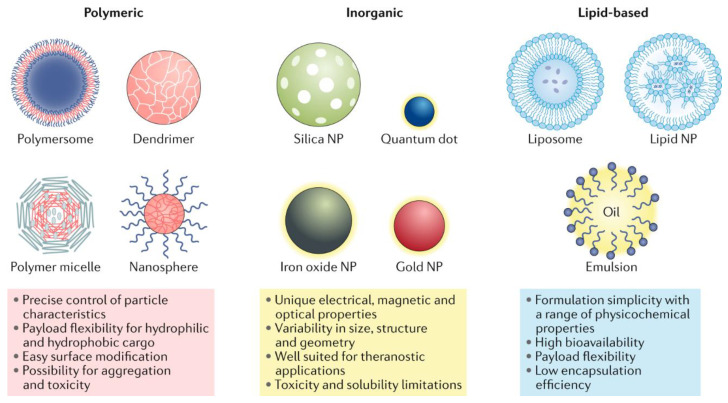
Each class of nanoparticle (NP) features multiple subclasses, with some of the most common highlighted here. Each class has numerous broad advantages and disadvantages regarding cargo, delivery and patient response. Reprints from: Mitchell, M.J., Billingsley, M.M., Haley, R.M. et al. Engineering precision nanoparticles for drug delivery. *Nat. Rev. Drug Discov.* **20**, 101–124 (2021) [4].

For various biomedical applications, drug delivery and imaging purposes, inorganic-based materials such as gold, iron, and silica have been employed to produce nanostructured materials. The different structures are shown below in Figure 1.

Iron oxide is the most studied material as FDA-approved nanomedicines [5]. At specific diameters (from 15 nm and no more than 100 nm), magnetic iron oxide NPs (MNPs), which are made up of magnetite (Fe_3_O_4_) or maghemite (Fe_2_O_3_), have proved to be effective as contrast agents, drug delivery vehicles, and thermal-based therapeutics [6]. This material is with a uniquely capable biomedical applications efficiency, such as diagnostics, imaging and photothermal therapies. The biocompatibility and stability are filling the niche of applications that requires properties impossible by organic materials. However, they are restricted for some clinical applications by low solubility and toxicity effects [6,7].

The distinctive feature of these nanoparticles is associated with their response to the action of an external magnetic field. In this sense, there is a great variety of materials from which MNPs can be obtained [8,9]:

- Pure transition metals: Fe, Co, Ni.
- Rare earth metals and compounds: Europium sulfide and oxide (EuS, EuO), Gd (gadolinium), Dy (dysprosium), Tb (terbium).
- Metal oxides: hematite (α-Fe_2_O_3_), maghemite (γ-Fe_2_O_3_), magnetite (Fe_3_O_4_), wustite (FeO), ferrites of various metals (BaFe_2_O_4_, MgFe_2_O_4_, CoFe_2_O_4_)
- Alloys: Fe-Co, Fe-Ni, Fe-Pt, Co-Pt.

The application of superparamagnetic iron oxide nanoparticles which act as an advanced platform for drug delivery, contrast agent in the image diagnostics and hyperthermia treatment for the cancer [10,11,12,13,14,15,16]. The magnetic nanoparticles are with broader degree of application and in the environmental science as well [17,18,19,20,21].

The intrinsic properties of SPIONs such as magnetic characteristics turns them such as a widely desired object of application in nonmedical applications. The superparamagnetic properties of these nanoparticles are broadly used for diagnosis, drug delivery and therapy. Likewise, theranostic multifunctional SPIONs brand them a proper dual agent for application in delivery and imaging diagnostics simultaneously. The key achievement in in vivo drug delivery application using SPIONs is the suitable magnetic field gradient.

The great interest and breadth in the fields of application of MNPs are related, among other characteristics, to their magnetic properties and the surface reactivity they possess as a consequence of the reduction in their size.

Between the mentioned elements and oxides and their combination able to assess magnetic properties, iron oxides, fundamentally magnetite and maghemite are the preferred not because of their distinctive properties regarding mainly to the superparamagnetic property but also their afoteric property allowing the change in the surface charge depending on the pH of the media. This characteristic extends the possibility to these oxides may be surface functionalized. In addition to the Fe characteristic of being a Lewis acid led to the additional ability to interact with several ligands by coordination bonds determining more stable and long-term links. In the specific biomedical area, the preference of these oxides is related to their biocompatibility since they may be metabolized as the endogen Fe and their intrinsic diagnostic and therapeutic capabilities.

### 1.2. Fundaments of SPIONs Applied to Biomedicine

In the last two decades, the interest regarding the application of MNPs, in particular iron oxides based (magnetite and maghemite), is growing due to the wide variety of applications they present in the biomedical field. Their use in cancer treatment as carriers for drug delivery and imaging contrast agents has long been reported. This is because of their distinctive performances based on their magnetic properties and nanoscale structure [22,23]. For the design of multi-functional MNPs, controlled surface engineering is critical for obtaining the required efficiency on the selected application [24]. The uses of MNPs as magnetic resonance imaging (MRI) agents for sensitive and precise diagnosis tools and synergistic combination with other imaging modalities were broadly explored. The recent progress in therapeutic applications, such as hyperthermia is discussed in Petropoulos’ work [25] and the available toxicity data of magnetic nanoparticles concerning in vitro and in vivo biomedical applications are addressed [26].

Another biomedical application is related to the transport of drugs and their accumulation at a specific site. Due to the magnetic core, MNPs offer the possibility of being guided by applying an external magnet [25,27]. Once located where needed, the release of drugs can occur avoiding, or at least reducing potential side effects. Those are, in general, of significance when administered systemically. To achieve specificity in their action once localized, it is necessary that the MNPs interact selectively with targets of biological interest such as cells or tissues of the organism. To do this, the surface of MNPs is commonly functionalized with different biomolecules, which will depend on the type of pathology that is to be addressed [28,29,30].

The ligands incorporated onto the surface of the MNPs recognize affinity molecules expressed or overexpressed exclusively in a certain type of cells. In this way, it is possible to achieve interactions (receptor-ligand or antigen-antibody, for example) that are useful for magnetically marking certain types of cells, such as tumor cells [30,31] and visualize them using MRI. Likewise, MNPs can transport drugs or molecules that are capable of acting on target cells, exerting a therapeutic action. This ideal model of mono- or multifunctionalized MNPs constitutes an effective strategy to reach and act on the desired cells [32].

**Figure 2:**
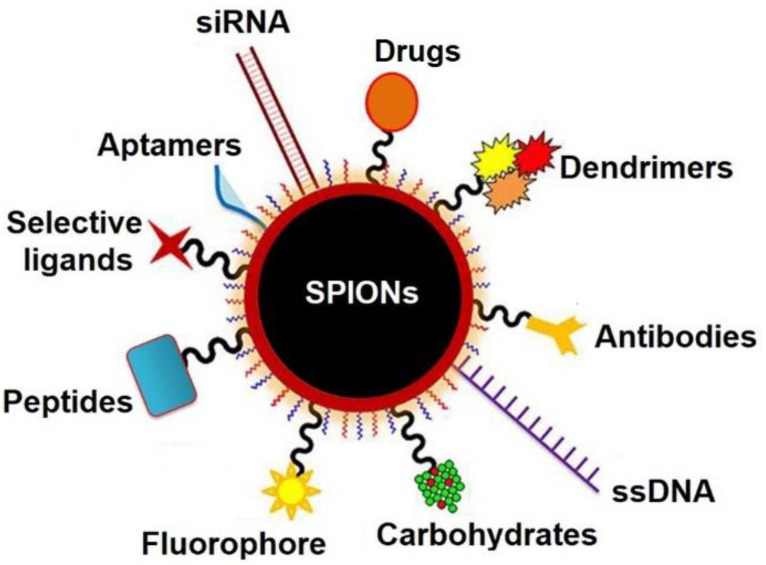
Representation of possible MNP functionalizers able to confer the theranostic identity.

Multifunctionalized MNPs such as those described in the previous paragraph have been proposed as theranostics. This term was first used by PharmaNetics President and CEO John Funkhouser in 2002 who defined it as a system with the ability to combine imaging and therapy modalities in a single device [33]. Figure 2 illustrates the concept of multifunctional MNPs or magnetic theranostic agent

In this review, we discuss the multiple biomedical applications of MNPs, emphasizing improved and specific capabilities on various diagnostic and therapeutic issues. We focus on advances in nanoparticle design, which can improve efficacy in general applications for theranostics efficiency-oriented to the cancer treatment. The combination of multitherapy and multimodal abilities on MNPs is addressed, highlighting the biological tests required for the evaluation of these nanosystems. These emerging topics and advances in engineering MNPs for very focused applications are of particular meaning as novel breaks arise for the clinical translation of MNP-based therapies in cancer and nanomedicine. As part of the information provided for this review, we have added original data reached in our Labs, as will be specified in later sections.

### 1.3. Structural Features and Surface Chemistry Linked towards the Biomedical Application

Size and morphology play an essential role in the depth of tissue penetration. Spherical SPIONs (<10 nm) are used to have the highest penetration and retention, compared to larger ones. Naud et al. have noted the importance of the size and morphology of SPIONs for cancer treatment [34]. The morphology and particle size must be appropriately chosen to address specific biomedical challenges. The particle size should be small enough to pass through capillaries in transdermal drug delivery (TDD) via intravenous injection and sufficiently large to be manipulated via magneto-mechanical actuation. For instance, single spherical particles (hydrodynamic diameter of 10−20 nm) are specifically suitable for injections into biological samples, but their low magnetic volume limits the magneto-mechanical effects. Clusters of SPIONs within the range of 100 nm to a few micrometers could address this problem. In this regard, Wilhelm et al. evaluated the role of particle size and morphology in the transport mechanism of blood [35]. The delivery efficiency versus particle physical properties indicated that <1% of particles were delivered, which was ascribed to fundamental limitations such as filtering and adsorption by other organs.

Increasing the ratio of surface atoms to core atoms is the main reason why the surface structure has a substantial impact on the properties of SPIONs. Surface effects of SPIONs are associated with charge transfer, lattice relaxation, oxidation, surface spin disorder (resulting in spin-glass-like behavior), and spin canting. Such effects could trigger several observed magnetic properties of SPIONs, including magnetic anisotropy enhancement and saturation magnetization reduction. The outermost surface layer of SPIONs defines many of their properties. Noncoated SPIONs have a tendency to aggregate, and after injection into the bloodstream, their surfaces are coated with plasma proteins, which is related to opsonization. To prevent or significantly reduce this immune process, the hydrophilic coating is preferentially used. As for SPION aggregation, this is reduced through electrostatic interactions or steric hindrance, wherein the surface charge of coating is essential.

The aggregation tendency of SPIONs and their ability to bind with serum protein was linked to the surface charge of SPIONs in colloidal suspensions. Even though most of the SPIONs are negatively charged, a more potent ability to bind with serum proteins is reported in positively charged SPIONs. The coating can block the release of Fe ions and dictate their interactions with biological entities, thereby significantly affecting their toxicity [36]. To target specific cells or tissues, the surface of SPIONs are modified by attaching various ligands, functional groups, or antibodies, which renders them more biocompatible.

The structural features and surface response of SPIONs [37], having good biodegradability, tissue penetration and reduced toxicity, showing good qualities in pharmacokinetics as magneto-mechanical actuators, MRI tracers and contrast agents, are considered good active agents in carcinoma treatments [38]. In fact, SPION is referred to 20–150 nm spherical magnetite Fe_3_O and maghemite γ-Fe_2_O_3_ crystals [36]. Size and morphology are determining for the level of tissue penetration—the smaller ones (<30 nm) are showing the highest cellular uptake and best retention [39], but quickly absorbed via pinocytosis, and bigger ones (~100 nm) have the best magneto-mechanical guiding selectivity [40], but strongly phagocytosed. This effective size of particles is critically defined by their ability to bind with serum proteins, forming stable aggregates (so-called zeta potential) [41]. The optimization of the size, shape, composition and surface modification of SPIONs will make them suitable for clinical applications. One of the aspects artificial engineering is focusing on is modifying the active surface of SPIONs, for achieving hydrophilicity. The clinical use of SPIONs, optimization of the composition, size, shape, and, most importantly, surface modification of SPIONs is necessary. However, it is primarily dependent on the artificial engineering of the surface of SPIONs, and on the miniaturization strategies to produce SPIONs at large scale suitable for clinical applications.

This involves capping SPIONs with organic acids (citric acid [42] and oleic acid [43,44]) or coating them with biocompatible hydrophilic polymers (poly(ethylene glycol) (PEG) [45,46,47,48,49] poly(vinyl alcohol) (PVA) [50,51], polyvinyl pyrrolidine (PVP) [52], polysaccharides, such as chitosan or dextran [53,54,55,56], while noncoated SPIONs have a tendency to aggregate in the bloodstream after intravenous injection. PEG-SPIONs show prolonged half-life time of SPIONs and reduce their cellular uptake. PVA-SPIONs show meager nonselective cellular uptake and do not stop blood flow in narrow vessels. PVP-SPIONs have also improved half-life time and do not affect cells’ vitality. Chitosan- and dextran-coated SPIONs are also well described, contrary to other types of coating, such as albumins, citrates, proteins and inorganic compounds were proposed, but their advantages are still not well observed [57,58]. The detection and accumulation of nanoparticles are used in a wide range of tissues—glioma (iron oxide NPs) [59], hepatocellular carcinoma (SPIONs hyperthermia) [60], breast (NP immunotherapy) [61] ovarian and cervical cancer (SPIONs) [39].

In conclusion, a vast variety of nanoparticles is known for now—ultrasmall polymer-lipid hybrid NPs, dendrimers, liposomes, quantum dots, carbon nanotubes, gold NPs, iron oxide NPs, etc. [62], but an experimental evaluation of each different carcinoma therapy has not yet been performed. Wilhelm et al. gathered data to associate nanoparticle physicochemical parameters, tumor models and cancer types with the low delivery efficiency via multivariate analysis [63]. Kar et al. constructed a predictive model based on regression nano quantitative structure-activity relationship (nano-QSAR), to bind cellular uptake to physical, chemical and structural NPs properties [64].

The afore discussion puts in relevance the need to orient the synthetic procedures to the required properties of MNPs.

## 2. MNPs for Cancer Therapy and Diagnosis

According to global cancer observatory data, one of the biggest scourges for society is cancer diseases, where 45% of the cancer sick patients died. The highest estimated mortality rates are in lung and breast cancer. Cancer nanomedicine has developed various items that have improved cancer care as a result of significant research and preclinical expenditure. Nonetheless, because the number of licensed drugs and their clinical performance is low, there is a view that cancer nanomedicine “has not lived up to its promise”.

**Figure 3:**
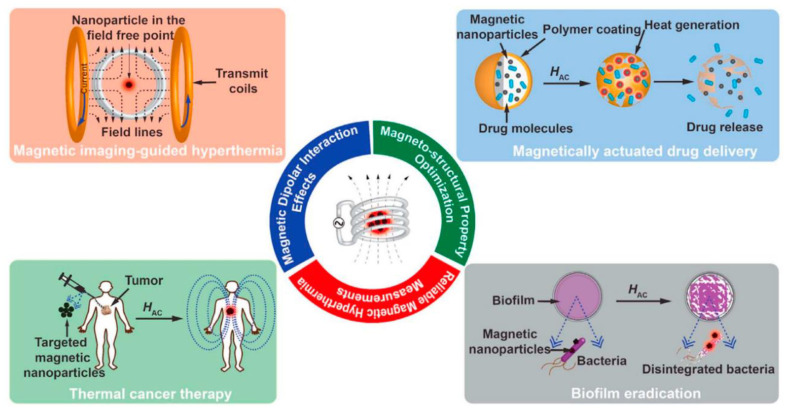
Schematic representation of multifunctional capabilities of magnetic nanoparticles for various types of biomedical applications, reproduced from [65], Elsevier, 2011.

Magnetic nanoparticles are a proper biomedical platform with multifunctionality applications focused on different uses with controllable parameters affecting the effectiveness of the treatment. are shown in Figure 3 [65].

Many of these studies ignore the long clinical history of iron oxide nanoparticles and the numerous clinical products that have resulted from their use. Although conventional treatments, such as surgery in the case of solid tumors, antitumor drugs, and radiation save lives, they still have enormous adverse side effects [66]. Over the past two decades, magnetic nanoparticles have started being utilized in biomedicine [67,68]. They appeared as a new therapeutic alternative in magnetic resonance imaging and the treatment of cancer [69]. Due to their small size, they can readily interact with biomolecules both at the surface and inside cells, providing magneto-mechanical actuation of cell surface receptors, tissue marking, targeted drug delivery, and triggered the release and magnetic hyperthermia [9,18,70]. Cancer diagnostics and cancer hyperthermia are only a few of the FDA-approved uses for iron oxide nanoparticles. We review the clinical experience with systemic liposomal drug delivery and parenteral therapy of iron deficiency anemia (IDA) with iron oxide nanoparticles. We note that the clinical success of injectable iron exploits the inherent interaction between nanoparticles and the (innate) immune system, which designers of liposomal drug delivery seek to avoid. Magnetic fluid hyperthermia, a cancer therapy that harnesses magnetic hysteresis heating is approved for treating humans only with iron oxide nanoparticles. Despite its successful demonstration to enhance overall survival in clinical trials, this nanotechnology-based thermal medicine struggles to establish a clinical presence. The extensive clinical experience with iron oxide nanoparticles [71,72] promoted new and exciting research points to surprising immune-modulating potential. Recent data demonstrate that the interactions between immune cells and iron oxide nanoparticles can induce antitumor immune responses: these present new and exciting opportunities to explore additional applications with this venerable technology. Clinical applications of iron oxide nanoparticles present poignant case studies of cancer nanomedicine possibilities, complexities, and challenges. They also illustrate the need for revised paradigms and multidisciplinary approaches to develop and translate nanomedicines into clinical cancer care.

Strong interactions of nanoparticles with host immune systems were recognized early and cancer nanomedicine development has tried to minimize these interactions in order to enhance drug delivery to solid tumors [72,73,74]. In the work of Choi et al. [75] stressed the advantages of nanomedicines with different applications as dendrimers, nanocrystals, emulsions, liposomes, solid lipid nanoparticles, micelles, and polymeric nanoparticles. The authors stressed the advantages of nanomedicines over conventional medicines and classifies as efficacy, safety, physicochemical properties, and pharmacokinetic/pharmacodynamic profiles of pharmaceutical ingredients. The enrolments of advanced nanomaterials and bioactive molecules into nanoparticle-based systems demonstrates the synergistic advantages of nanocomplexes as compared to the individual components for a drug delivery application [76,77] and cancer treatment platforms [78,79].

Modulating these interactions in the context of disease and the altered immune microenvironment is an interesting area of research. A complete understanding of nanoparticle interactions with immune cells remains a critical gap in knowledge impeding progress to develop effective cancer nanomedicines. Obtaining this complete understanding is a significant challenge since minor differences between nanoparticles have a significant impact on immune interactions.

Immune reaction to iron oxide nanoparticles can depend considerably on their size, route of administration, dose, raw materials, coating, etc. The reactions can be hypersensitivity, inflammation, immunosuppression, immunostimulation, complement activation, or a combination [80,81]. Iron oxide nanoparticles elicit host immune responses that release cytokines and chemokines in the blood. In mouse models, magnetite (Fe_3_O_4_) nanoparticles having diameter 5–8 nm induced inflammatory reactions post intratracheal instillation measured by a dose-dependent increase of pro-inflammatory cytokines IL-1, TNF-α, and IL-6 in bronchoalveolar lavage fluid (BAL), and in blood [82]. Carboxydextran coated iron oxide nanoparticles (Resovist^®^) attenuated OVA-specific IgG1 and IgG2a and reduced IFN-γ and IL-4 production by splenocytes in OVA-sensitized BALB/C mice [83].

On the other hand, complement activation occurred with dextran-coated iron oxide nanoparticles [84]. Due to iron oxide’s inherent MRI contrast property, many studies are conducted by imaging using these nanoparticles. It is particularly noteworthy that iron oxide nanoparticles can be helpful to assess inflammatory disease progression, often with MRI [85]. In other words, depending on the model and disease context, exposure to iron oxide nanoparticles can be immune-stimulating or immune-suppressing. Unique physical and chemical properties arise from the high surface-to-volume ratio of nanometer-scale materials. Thus, it is likely that this aspect of nanoparticles is also responsible for much of the nanoparticle-immune cell interactions, making the nanoparticle coating particularly important. Depending on the layer, nanoparticles can present different features to immune cells, generating different cellular responses, particularly when modified to include a protein or ligand for active targeting [86]. Korangath et al. demonstrate that a humanized monoclonal antibody on the surface of iron oxide nanoparticles led to significant retention in the tumor microenvironment via capture by a resident (tumor-associated) innate immune cells.

The uptake of nanoparticles by host immune cells altered the tumor microenvironment leading to growth inhibition through T cell activation. In a series of elegant studies, Lo et al. demonstrated that anti-CD3 antibody-coated nanoparticles enhance T cell receptor crosslinking on effector T cells, which is an activation signal and can improve the efficacy of vaccines and immunotherapy. Subsequently, Kosmides et al. demonstrated that antibodies conjugated to the surface of nanoparticles could activate CD8+ T cells [87]. Here they used an antibody against immunosuppressive PD-L1 antibody and a co-stimulatory agonist 4-1BB antibody conjugated to iron oxide dextran-coated nanoparticles and injected directly into tumors.

### 2.1. Photothermal (PTT) and Photodynamic Therapy (PDT)

Photothermal therapy (PTT) uses light-absorbing agents, which thermally damage tumors when irradiated with NIR laser, providing deep tissue penetration with minimum nonselective cell death [88]. Photodynamic therapy by itself uses agents, generating singlet oxygen as a destructor [89]. Usually, the employed radiation belongs to the near-infrared radiation range. At this range, the absorption and scattering of the radiation by the body is minimal. Thus, tissues are almost transparent. To improve the efficacy and selectivity of the energy-to-heat transduction, a light-absorbing material, the photothermal agent, must be introduced into the tumor. At present, a vast array of compounds is available as photothermal agents. Among the substances used as photothermal agents, magnetic nanoparticles are an excellent photothermal agent in treating tumors. Employing raw magnetic nanoparticles leads to the limitation that their molar absorption coefficient is in the near-infrared region is low. The controlled clustering of the nanoparticles can solve this drawback. In such conditions, the absorption of the indicated radiation is higher and the conversion of energy in heat is more efficient than in individual nanoparticles.

The iron oxide nanoparticles heating efficiency can be elevated when we combined the infrared radiation with an alternating magnetic field [90]. The way of such a combinations of the both methods could release a drug attached to the nanoparticles in a precise manner. The release of drug seems to be a capable implementation for chemo-phototherapy.

PDT functions as a cancer treatment strategy based on the generation of cytotoxic ROS, by means of photosensitizers (e.g., porphyrin, chlorine e6, indocyanine green, and Rose Bengal) under specific light irradiation.

### 2.2. Hyperthermia

As mentioned earlier, hyperthermia is a cancer therapy whose objective is to raise the local tumor temperature to either kill cancer cells or sensitize them to other treatments [91]. Mild hyperthermia keeps the temperature between 39–41 °C and severe hyperthermia—over 45 °C, where tumor annihilation ensues. There are plenty of side effects, of course [92]. Efficient use of magnetic hyperthermia in clinical cancer treatment requires biocompatible magnetic nanoparticles with improved heating capabilities. Various synthesis routes have been explored for the production of IOMNPs with enhanced magnetic properties [93]. In this study, we are also focusing the reader’s attention on an iron oxide nanoparticle cluster. The self-assembly of nanoparticles into three-dimensional clusters has been popular for biomedical applications.

The iron oxide nanoclusters have been synthesized utilizing individual Fe_3_O_4_ nanoparticles with different sizes as building blocks. Individual Fe_3_O_4_ nanoparticles were encapsulated in an oil in water emulsion by hydrophobic interactions between cetyltrimethylammonium bromide (CTAB) and the nanoparticle’s surface aliphatic capping agents. Time, temperature, and CTAB concentration were three important parameters in determining the size, form, and collective behavior of the clusters. The clusters’ magnetic hyperthermia behavior has also been investigated.

According to the data, both clustering and the size of the main nanoparticles improve the Specific Absorption Rate (SAR) values. Due to the higher connection between the clusters’ magnetic moments compared to the individual nanoparticles, the dipole interactions inside the clusters boost their heating performance for both types of clusters.

As a result, clusters are preferable for biomedical applications such as magnetic hyperthermia due to their collective behavior.

Due to the collective magnetic behavior arising from multiple Fe_3_O_4_ nanoparticles assembled in each cluster, hyperthermia measurements of different sizes of Fe_3_O_4_ nanoparticles and their clusters demonstrate the Specific Absorption Rate (SAR) for clusters of each size is higher than the SAR for individual nanoparticles.

Interparticle interactions can lead to collective magnetic behavior in these 3D spherical assemblies of Fe_3_O_4_ nanoparticles, comparable to cluster-assembled magnetic nanoparticle films and close-packed nanoparticle arrays [94].

According to findings, SAR for clusters with more significant individual nanoparticles is also substantially more outstanding than SAR for groups with smaller nanoparticles.

This can be explained by considering the effect of size in decreasing the spin canting effect in nanoparticles.

## 3. Nanomaterials for Smart Therapeutic Multifunctional and Multimodal Systems Based on SPIONs Combined with Other Nanomaterials

The fabrication of structures composed of SPIONs combined with other functional moieties have emerged as a suitable strategy for obtaining magnetic theranostics. These nanosystems cover a range of diagnostic and therapeutic applications in a unique dispositive.

To even improve the efficiency of these systems, the occurrence of specific ligands or functional abilities are required. This may be achieved by the structural and/or surface modification of SPIONS with other compounds. Although the open literature includes a wide amount of information in this regard, here in this contribution we will focus on the novelest ones aiming to show the synergic properties that may be achieved by the combination with regards to the therapeutic and diagnostic capabilities.

Therefore, we refer to some inorganic compounds such as gold and gadolinium; and between the organic ones we center the attention in lipidic–magnetic nanostructures as a field slightly studied and reported in the existing literature

### 3.1. Multitherapy Magnetic-Based Nanotheranostics

The medicine paradigm has now turned from monotherapy to multitherapy procedures, enhancing treatment efficiency by combining diverse capabilities of diagnostic and therapy in a unique dispositive. From this point of view, the design of MNP based nanodispositives able to cover several therapeutic capabilities increase the success of the treatment allowing the implementation of the most suitable therapeutic ways depending on the pathology to fight to.

In the case of tumoral diseases de combination of magnetic hyperthermia with magnetic guided chemotherapy (by drugs loading), PTT and/or PDT, as shown in earlier section, may derive in a synergic capability and higher efficiency to the treatment of some kinds of tumors [95], To reach this goal the structure of surface of SPIONs have necessary to be functionalized with the appropriate active agents. Therefore, in order to obtain the complex design work, it requires multitherapeutic systems with specific properties regarding the desired application [96].

As above mentioned, here we selected the less explored SPIONs’s modifiers aimed to determine the multitherapeutic capability.

#### 3.1.1. Lipidic-Magnetic Nanoparticles

Among the vast volume of information regarding the use of MNPs in the biomedical area, it has been well documented that the application of bare MNPs tends to be ineffective drug carriers due to some limitations in drug loading, release rates and retention time in the bloodstream [97]. Furthermore, it was reported that MNPs could suffer aggregation once inside a biological system affects their colloidal stability and cause toxicity [97]. These drawbacks have limited the application of magnetic nanoparticles on a commercial level. To overcome these problems the magnetic nanoformulations can be synthesized by combining them with biocompatible materials such as lipid-based nanocarriers [98]. Studies concerning the combination of MNPs and liposomes showed protection against the body immune system, increasing their stability in the body fluid, overcoming its acute agglomeration and obtention of more biocompatible nanocarrier [98]. At the same time, it was observed that the combination of MNPs with liposomes enhances magnetic resonance imaging (MRI) contrast [99]. Higher sensitivity to the magnetic field due to the highest magnetic permeability among the iron oxides [100] and the ability to perform photocatalytic reactions was observed [101]. Furthermore, the combination of magnetic nanoformulations with micelles has been reported, finding great stability and biocompatibility, magnetophoretic control of the nanoformulations and enhancement cellular uptake by cancer cells in vitro [102].

In this concern, preliminary studies have been recently developed in Lassalle´s research group. Nanostructured lipid carriers (NLCs) loaded with Diclofenac (a commonly used Non-steroidal anti-inflammatory drugs (NSAID)) were decorated with magnetic nanoparticles to assess a lipidic-magnetic nanosystem able to transport and deliver active principles such as Diclofenac. NLCs differ from the solid lipidic nanoparticles (SLNs) in the physical state of the lipids that compose them. While SLNs are composed of solid lipids, the components of the NLCs include mixtures of solid lipids and liquid lipids (oils). Due to the presence of liquid in NLCs structure, the crystalline grid of the solid is disrupted and, as a result, the load capacity of the carrier system is significantly improved. The goal is to enhance the carrier performance by combining the biocompatibility properties of the lipids with the ability to target provided by the magnetic phase.

The NLCs coating (composed of 75% (*w/w*) ethyl oleate and 25% (*w/w*) soy lecithin as lipidic phase) was performed using MNPs aqueous dispersion containing 0.5 mg of MNPs per 1 mL. Different volumes of the magnetic distribution (ranging from 0.128 to 1.28 mL) were mixed with 1 mL of lipid nanoparticles suspension during 360 min under magnetic stirring at room temperature. It is essential to highlight that the MNPs employed in this procedure were composed of magnetite as core and chitosan as coating according to the method reported in Nicolas et al. [103].

The interaction between the nanoparticles may be explained by considering that both, magnetic and lipidic nanoparticles, have an intense surface charge with opposite signs. This justification may be evidenced by the zeta potential data recorded for different batches containing increasing ratios of MNPs/NLCs. The cited data is included in Table 1.

The original data included in Table 1 reveal that the MNPs/NLCs, as expected, realize a partial neutralization of the surface charge when these systems are compared with their precursors (raw MNPs and NLCs). This fact offers the possibility to modify the surface charge defining versatile nanosystems able to be loaded with therapeutic agents of different nature.

Based on the physicochemical properties, lipidic-magnetic nanosystems appear as promising tools in the design of theranostics agents destined for cancer and other critical pathologies.

#### 3.1.2. Magnetic Inorganic Nanomaterials

One of the most attractive elements combined with SPIONs is Au. Especially in oncological diseases treatment, the combination of magnetic nanoparticles with Au has opened novel perspectives to attain more efficient and diverse therapies for different kinds of tumors. Briefly, Au NPs may act as therapeutic approaches, including phototherapy (PTT), photodynamic (PDT), radiotherapy (RT), chemotherapy, and hyperthermia. As many of these practices are low or non-invasive, the incidence of Au-based nanomaterials in clinical assays is actually in growth [104].

In most cases, thermal ablation of the tumor is induced using a laser beam converted by Au NPs into heat, promoting a series of biological phenomena, including protein denaturation, apoptosis, and necrosis. This leads the tumor cell death [105]. The main limitation of this therapeutic strategy is the low penetration level of the light inside the tissues, which made this technique only helpful in treating surface tumors [106]. In recent years, the design of combined Au-SPION nanosystems has attracted significant attention due to the possibility of overcoming the mentioned Au NPs limitations. Some authors demonstrated the potential of Au-coated SPIONS with high NIR light absorption as a candidate for cancer photothermal therapy [104].

RT is a crucial therapeutic mode for treating nearly 50% of cancer patients [107]. Recently, much interest has been raised in applying AuMNPs as radiosensitizers in RT for cancer, with particular attention to solid hypoxic tumors due to various advantages of gold nanostructures, for example, high absorption and efficiency in generating secondary electrons under g-ray or X-ray irradiation [68]. It has been shown that Au MNPs with different sizes and shapes can significantly improve the effectiveness of cancer therapy mediated by RT and HT. Based on the efficiency of the reported results, the composition of Au- iron oxide nanocomposites is a crucial factor in regulating their physicochemical properties conditioning their performance concerning multimodal imaging and therapy [107].

The incorporation of Gd moieties to SPIONS nanostructures is known (as will see in the next section) that significantly improves the possibilities of such nanosystems to generate dual contrast effect in diagnostic mediated by magnetic resonance image (RMI). However, its impact on the therapeutic capabilities of Gd doped or surface modifying MNPs has been less explored. The existent works consider the surface functionalization of iron oxides nanoparticles with Gd oxide. For instance, Szpak et.al. Have prepared dual-mode MRI contrast agents consisting of superparamagnetic iron oxide nanoparticle (SPION) cores and gadolinium ions associated with ionic chitosan protecting layer. Gadolinium ions were introduced into the coating layer via direct complex formation on the surface of the nanoparticles, covalent attachment, or electrostatically driven deposition of the preformed Gd complex. Although these nanoplatforms are highly promising regarding their RMI diagnostic capabilities, they lack the efficiency to induce magnetic HT due to the difference in the magnetic behavior of each component of the nanosystem [106]. Gd(III) is known to oppose the net magnetic moment of Fe(III)/Fe(II) in oxides, reducing magnetization. The therapeutic action may be provided in this case by loading a specific drug.

An alternative strategy to conserve the HT ability is the incorporation of Gd as a dopant of the iron oxide structure. Recent articles lead to the synthesis of Gd-doped Fe_3_O_4_ nanoparticles that can act as effective MHTagents [108]. Some studies demonstrate higher SAR values for Gd-doped Fe_3_O_4_ nanoparticles than the reported values for undoped samples. Naik et al. published the preparation of Gd0.075Fe2.925O4 nanoparticles by coprecipitation method. These authors revealed that the Gd doping on the Fe_3_O_4_ nanoparticles affects their morphology and magnetic properties. Still, the magnetic hyperthermia efficiency of the samples was about the same within the experimental uncertainties. Considering the existing literature, it may infer that the Gd incorporated dose resulted as a critical factor in assuring the HT therapeutic capability of these dual nanosystems [109].

### 3.2. Multimodal Magnetic-Based Contrast Agents

**Figure 4:**
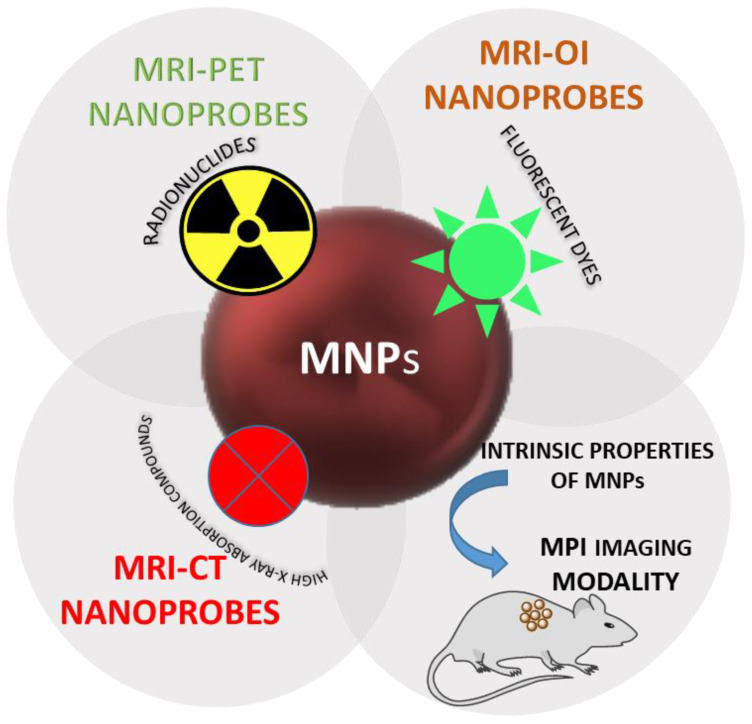
Representation of the capabilities of MNPs to impart multiple diagnostic tools.

Imaging techniques are usually classified as morphological and molecular. In the former are included modalities that provide anatomical information such as Magnetic Resonance Imaging (MRI), computed tomography (CT) and ultrasound imaging (US). Molecular imaging techniques are positron emission tomography (PET), single-photon emission CT (SPECT) and optical imaging which provide information at the cellular and molecular levels. The Figure 4 illustrates the concept of multimodal contrast magnetic based contrast agents. These techniques not only allow the visualization of specific events in the progress of diseases but also can be useful for monitoring therapeutics treatments. The developments of contrast agents and imaging techniques allow the inclusion of MRI and CT in molecular imaging [110]. Detailed information is highly needed for a precise diagnosis of diseases. This information could be challenging to obtain with a single imaging modality since they have advantages and disadvantages. For example, MRI and CT have a special high resolution but poor sensitivity. On the other hand, PET has high sensitivity but the physics of positron decay and the process of annihilation limits its spatial resolution [111,112]. The single imaging modalities, multimodal imaging has emerged as a way to obtain an accurate disease diagnosis. To succeed in molecular imaging, it is essential to develop an adequate contrast agent (CA). Nanoparticles are promising candidates for CA since surface modification can be carried out with different targeting molecules and present a prolonged circulation time. In this way, enhanced affinity for biomolecules or cells is achieved. Among the different kinds of nanoparticles, iron oxide magnetic nanoparticles have attracted particular attention. They have been used as a contrast agent for MRI due to their superparamagnetic behavior. Some contrast agents based on iron oxide have been in the market, although the majority was withdrawn, mainly for marketing reasons [113]. Furthermore, easy conjugation with biological molecules and a wide range of imaging moieties make them an essential platform for the design of multimodal imaging nanoprobes [114].

In the following sections, the modification of iron oxide magnetic nanoparticles to obtain potential candidates for multimodal imaging will be discussed. Furthermore, it will be seen how the inherent properties of magnetic nanoparticles could lead to new molecular imaging such as Magnetic Particle Imaging (MPI).

#### 3.2.1. MRI-Positron Emission Tomography (PET)

Positron emission tomography visualized and quantified positron-emitting radionuclides that are administrated to patients. A wide range of radiotracers provides images related to physiological conditions [112]. Since this technique has high sensitivity but poor resolution, the combination with MRI is synergistic due to the excellent soft-tissue contrast and high resolution of MRI.

Multimodal MRI-PET contrast agents can be obtained by modifying magnetic nanoparticles with radionuclides. The design of nanoprobes that fulfill the requirements of nuclear and magnetic images is not an easy task. The main problem is the doses of radioisotopes that are usually administered in PET and the doses of contrast agents required for MRI. The difference in amounts is about seven orders of magnitude. So that nanoprobes have to have a good performance in MRI and PET and have to solve the differences in doses for a given target. [115].

Magnetic nanoparticles are conjugated through chelators (usual macrocycles) that form stable complexes with radioisotopes. The most common macrocyclic chelators used are 1,4,7,10-tetraazacyclododecane-1,4,7,10-tetraacetic acid (DOTA) and the one of 1,4,7-triazacyclononane-1,4,7-tri acetic acid known (NOTA) [116].

Radiolabeled magnetic nanoparticles have been modified to reach a determined pathology. For example, Hajiramezanali et al., conjugated 68 Ga to trimethyl chitosan-coated magnetite using the agent chelator DOTA and then changed it with a bombesin derivative. This compound has a high affinity for gastrin-releasing peptide, a receptor overexpress in breast tumors [117]. The authors evaluated the capacity of the radiolabeled magnetic nanoparticles to detect breast cancer in mice by both MRI and PET techniques. Biodistribution assays showed that tumor uptake of 68 Gd-labeled magnetic nanoparticles was increased by time, reaching a percentage of 2,27% of the injected dose after 120 min. The authors concluded that the synthesized nanoparticles are potential tools for some cancer detection through dual-modality MRI/PET.

The introduction of radionuclides through chelators presents some difficulties, as the instability of nanoparticles in the conditions necessary for the molecular chelation of radioisotopes. Furthermore, chelators can be stripped in vivo from the surface of nanoparticles so that the images and biodistribution studies will not correspond to the actual distribution [118]. For that reason, chelators free radiolabeling methods have been developed to modify nanoparticles with radioisotopes. Madru et al. incorporated 64Cu to amino-PEG coated iron oxide nanoparticles (64Cu-SPIONs) in a simple way with a high label efficiency (97%). 64Cu-SPIONS demonstrated to be stable in vitro and in vivo studies up to 24 h. The ability of the synthesized agent to target sentinel lymph nodes (SLNs) was evaluated in vivo by PET-MRI images. Authors found that Both techniques visualized sLNs with reasonable accuracy up to 24 h of the intradermal injection. PET-MRI images were more informative than each technique separately [119].

The use of chelators different from the traditional ones has been evaluated to overcome the problem of the in vivo release of radioisotopes. Thomas et al. studied the use of MANOTA as a chelator of 64Cu^2+^ since this radionuclide has been found to release from DOTA in vivo [116]. The obtained magnetic nanoparticles showed good stability in suspension and high in vivo contrast by both PET and MRI, while no cytotoxicity was observed in in vitro tests.

#### 3.2.2. MRI-Optical Imaging

Optical imaging (OI) is a technique of high sensitivity but poor special resolution. So, the integration of OI with MRI would offer a modality of high sensitivity and high special resolution.

To construct dual contrast agents for MRI-OI a common strategy is to conjugate fluorescent molecules to magnetic nanoparticles [120]. This strategy was used to obtain nanosystems suitable for the diagnosis through MRI and optical imaging of different diseases. For example, DMSA-magnetite was modified with the fluorescent dye Cy5.5 and polyclonal antibody profilin- I to image atherosclerotic plaque [121]. Profilin-I was found to be overexpressed in the arterial wall of atherosclerotic plaque. Fluorescence and MRI images were performed in apoE-/- mice divided into three groups: one fed with a standard laboratory diet, the other fed with high fat and cholesterol diet and a third group fed with a high-fat diet and treated with atorvastatin. The results showed that not only the modified nanoparticles could improve the molecular diagnosis of atherosclerotic plaque by a dual-modality but also it could demonstrate the effects of atherosclerotic treatments.

Nanoprobes for dual MRI-OI were also designed to target tissues involved in cancer disease. For example, PEG-modified magnetite was conjugated with Cy5.5 and the cyclopeptide GX1 to image angiogenesis in gastric cancer [122]. MRI images obtained 8 and 12 h after injection showed a distribution of nanoparticles consistent with the formation of new vessels. Fluorescence intensity was higher in tumors than in all organs except the liver [123].

The conjugation of cross-linked magnetite with folate (to target folate receptor, an overexpress receptors in cancer cells) and Cy5.5 dye [124] KB cells (human nasopharyngeal cell line) were implanted in mice. Two hours after postinjection, MRI images showed that the tumor area became noticeable darkened because of the contrast T2 ability of magnetic nanoparticles and a high accumulation of them in the tumor area. Fluorescence images were taken 3 h after postinjection and confirmed the assembly of the nanoparticles in the tumor, although nanoparticles also accumulate in the liver, kidney, and spleen. The authors proposed that the selective binding to folate receptors and the enhanced permeation and retention (EPR) effect (this effect is described in Section 4.1 and in Figure 4) are responsible for this high accumulation.

#### 3.2.3. MRI and CT

Computer tomography (CT) and MRI are the most common imaging techniques to provide anatomical information with diagnostic intentions. CT has high resolution and allows the formation of a 3D image easily. Furthermore, it gives information on hard tissues (such as bones). However, it is difficult to detect slight changes in soft tissues with this technique. On the other hand, MRI presents good soft-tissue contrast, but it is not adequate for bony imaging structures. So that, the combination of MRI and CT could facilitate an accurate and reliable disease diagnosis [124].

Magnetite was combined with bismuth to achieve dual MRI-CT contrast agents [56]. Several studies refer to iron oxide-based hybrid nanoparticles for MRI-CT that combine the magnetic compound with gold. This material has remarkable properties for its use as a CT contrast agent, such as a high X-ray absorption coefficient [125]. An excellent job was carried out by Hu et al., who reported the synthesis of Fe_3_O_4_/Au modified with hyaluronic acid to target the CD44 receptor-overexpressing cancer cells [126]. A xenograft tumor model was used to evaluate the capacity of the nanoparticles to act as MRI and CT contrast agents. The tumor site became darker in MRI, while CT’s was brighter, indicating that the nanoprobe targeted the overexpress CD44 receptor.

Hemalatha et al., synthesized oleyl chitosan functionalized hybrid iron oxide/gold nanoparticles more recently. In vitro studies showed that the nanoparticles exhibited a decreased signal intensity depending on nanoparticle’s concentration (negative contrast) in MRI and X-ray attenuation in CT, which increases in a dose-dependent manner [127]. In vivo biodistribution studies were conducted in normal mice and both, CT and MRI showed the accumulation of the nanoparticles in the liver [128].

Another approach to obtain hybrid iron oxide/gold nanoparticles for dual imaging is the synthesis of gold nanoparticles decorated with magnetite. A chemical bond was achieved by reacting between aminated gold nanoparticles and carboxyl modified magnetite [129]. The evaluation of the imaging capacity as a dual contrast agent was also evaluated in a xenografted tumor model. The results obtained by CT and MRI were consistent and determined that the probe could act as a dual contrast agent.

#### 3.2.4. Magnetic Particle Imaging (MPI)

MPI is an emerging imaging modality that allows the visualization of magnetic nanoparticles with high sensitivity. In contrast with MRI, the obtained signal intensity corresponds directly with the concentration of the magnetic nanoparticles with no interference from surrounding tissues [130]. For that reason, MPI is a quantitative technique. For an accurate contrast in MPI, nanoparticles need to accumulate in pathological tissues at adequate levels so that their physicochemical properties are crucial for the success of the technique. If the core is too small, they are not suitable for MPI since the magnetic moment is not high enough. A diameter of 30 nm is ideal for this technique [131]. Phase purity is also essential. Amari et al. conjugated a brain cancer targeting peptide (lactoferrin) to magnetic nanoparticles. The authors also used an external magnet to increase the localization of nanoparticles in the tumor. They evaluated the accumulation of nanoparticles modified and non-modified with lactoferrin and found that the ones modified with the peptide were easily internalized and retained by the tumor [132].

The characteristics of MPI have made it appropriate for monitoring in vivo drug release, as was reported by Zhu et al. [133]. The authors loaded doxorubicin to a core-shell nanocomposite of magnetite clusters and PLGA (poly(lactide-co-glycolide acid). The shell (PLGA) degradates in mild acidic microenvironments, yielding the dissembling of the magnetic cluster while the drug is released. The magnetic cluster has a low signal in MPI, but this signal is enhanced during the degradation of the shell because of the rapid recovery of magnetic nanoparticles Brownian relaxation. As the degradation of the shell is correlated with the release of doxorubicin, a relation between this release and the change in the intensity of the MPI signal can be established. This was demonstrated in in vivo studies.

In summary, dual imaging modalities could facilitate an accurate and reliable diagnosis of diseases of high social impact, such as cancer. Nanoparticles, especially magnetic nanoparticles are invaluable tools for designing nanoprobes for dual imaging modality. Combining them with different bioimaging agents allows the formation of double-contrast agents that can be modified to target a specific region. Although biodistribution assays showed that iron oxide magnetic nanoparticles accumulate mainly in the liver and spleen in most articles, they were also collected in pathological tissue in a sufficient concentration for their visualization by different imaging modalities. Furthermore, the development of imaging techniques such as MPI opens new possibilities for the contribution of iron oxide magnetic nanoparticles in diagnosis.

## 4. Biological Findings of the Capacities of Magnetite-Based Magnetic Nanoparticles for the Management of Oncological Diseases

MNPs have been extensively studied for their different uses in biomedicine. Until now, it is known that they have low toxicity and reach optimal sizes that do not interfere with physiological or cellular functionality [11,18]. Even some magnetic nanosystems based in SPIONs are currently commercial for different applications in biomedicine, such as anemia treatment, detection of lymph node metastases, among others [113]. In mechanistic terms, it is necessary to internalize the SPIONs within the target organs/cells to exert their effects in many cases. The most known internalization pathway for SPIONs is endocytosis, after initial contact with the plasma membrane, an energy-consuming process. Later, endosomes are formed, vesicles with lipidic membranes that remain in the cell cytoplasm, carrying the nanoparticles. The endocytic pathway can be classified into phagocytosis, macropinocytosis, clathrin-mediated endocytosis, and caveolae-mediated endocytosis. The choice of cells for each will depend on the cell type and the nature of the SPIONs [134]. To enhance the biocompatibility, cell uptake and also to potentiate their performance in therapy and diagnostic, SPIONs can be functionalized with organic compounds, such as lipids, or can also include in their formulation other inorganic compounds as gadolinium, zinc oxide, gold, manganese, or radioisotopes, among others. This section will analyze potential composites that, in combination with magnetite-SPIONs, offer new possibilities in terms of multimodal diagnostic or multitherapy capabilities. Since these nanodevices are intended for future biomedical applications in cancer, an essential body of data must be obtained from in vitro and in vivo studies to move on to the clinical phases of the study. According to clinicaltrials.gov database, there are no studies in clinical phase involving the use of conjugated magnetite-based MNPs. Despite this, some completed unpublished research can be registered. For instance, magnetic nanoparticles have been tested for prostate cancer cells thermoablation. In another study denominated “Pre-Operative Nodal Staging of Thyroid Cancer Using USPIO MRI: Preliminary Study”, Ferumoxytol carbohydrate-coated superparamagnetic iron oxide nanoparticles, were used for MRI detection of lymph nodes of thyroid cancer. Ferumoxytol has been used in other terminated or withdrawn studies and is proposed in trials that are currently under recruitment (See clinicaltrials.gov). Therefore, it can be concluded that only simple or unconjugated iron oxide nanoparticles (in terms of additional materials described in this review article) are being proposed for clinical trials. Therefore, the combination of an iron oxide magnetic core with other materials such as lipids or inorganic materials is a therapeutic approach that has achieved only the preclinical or in vivo stage. This analysis provides an overview of the possible beneficial or adverse effects associated with these annexed materials that origins a nanosystem with a new physicochemical identity and, therefore, with different associated results at the biological level that need to be studied.

### 4.1. Magnetite Nanoparticles Combined with Lipid Compounds

Since a membrane of a phospholipid nature covers cells, the strategy of combining different nanosystems with lipids may have been one of the first approaches to increase their internalization. These nanocomposites have multiple characteristics. The magnetic component attributed to magnetite allows inducing targeting, detection, and hyperthermia phenomena. The latter is very advantageous since it produces the thermal ablation of tumor cells and allows the selective release of drugs at the target site. The lipid component, in this case, acts as a compatibilizer but is also capable of encapsulating lipophilic drugs in its matrix.

Furthermore, lipids are susceptible to chemical or pH stimuli [134,135]. The uptake of magnetite core-SPIONs by cells was markedly improved by coating with cationic lipids form micelles (L-SPIO). The L-SPIO average size (46 nm) was not influenced by the culture medium supplemented with fetal bovine serum did not influence the diameter and were subsequently incorporated by different cells of tumor origin such as HeLa (cervical cancer), PC-3 (prostate cancer), and Neuro-2a cells (neuroblastoma) without affecting their viability. Cells loaded with L-SPIO could be isolated by magnetic attraction for up to 10 days. In vivo assessment of L-SPIO performance revealed that the labeling of CRC cells allowed their neoplastic growth monitoring by RMI for up to 15 days after injection in mice. The inability of tumor cells to metabolize L-SPIO is notorious and becomes an advantage in future implementation for cancer multitherapy [136].

Magnetite nanoparticles embedded in PLGA were covered by a lipid monolayer and hybridized with PEG. This stable nanosystem was produced for camptothecin releasing by a magnetic field stimulus of remote radiofrequency. These nanoparticles internalize after one hour of incubation. A significant reduction in the viability of the mouse breast cancer cell line MT2 only when the experimental conditions involved drug-loaded nanoparticles and were simultaneously stimulated with radiofrequency. Otherwise, no toxicity was observed [137].

Magnetic solid lipid nanoparticles (size ≈ 180 nm) made of iron oxide cores embedded within a glyceryl trimyristate solid matrix. These nanoformulations proved in vitro to be hemocompatible. It was observed that the stimulation of these nanoparticles with a high frequency alternating magnetic field induces hyperthermia, which resulted in a marked decrease in the viability of HT29 cells derived from the human colon adenocarcinoma. Notably, no cytotoxicity was observed in nanoparticles-treated cells without a magnetic stimulus [14].

Another type of lipid encapsulation of SPIONs can be generated directly by uptake by cells or vesicles, called “natural lipid encapsulation,” distinguished from synthetic liposomes because they have natural lipid membranes, which increases their biocompatibility and durability. A challenge in this type of combination is to increase the amount and retention time of the SPIONs. On the other hand, this type of nanocomposites encapsulated in vesicles can be easily internalized by macrophages. From the MRI diagnostic capacity point of view, this is a disadvantage since they would give false positives in non-malignant or non-target cells [134].

Zang et al. reported a nanoprobe library of magnetite nanoparticles encapsulated in phospholipids and phospholipid-PEG copolymers combined with dialkylcarbocyanine dyes for multimodal magnetic resonance/fluorescence Imaging. U-87 MG cells from human glioblastoma, were incubated with various forms of these SPIONs, and it was shown that under a magnetic field stimulus, these nanoparticles enhanced the T2 contrast intensity. This effect was attributable to increased cellular uptake of the nanoparticles induced by magnetism. At the same time, these cells could be detected by fluorescence microscopy. They also conducted in vivo studies on mice bearing subcutaneous glioblastomas. The nanoprobes were injected via the tail vein, and MRI recorded the T2 contrast. It was possible to observe how the liver and the spleen, and later the kidney, were darkened by the presence of the SPIONs [138].

Near-infrared signal epifluorescence studies were carried out in parallel. Over time, the tumor became much brighter than the surroundings. Postmortem studies recorded large amounts of nanocomposites accumulated in the liver and spleen. Once again, the selectivity of this type of nanoplatforms is low and is subjected to the EPR effect [138]. In another investigation where the selectivity deficiency was solved, a similar approach was used for the non-invasive diagnosis of progressive hepatocellular cancer. In this case, the magnetite was combined with a fluorescent compound near-infrared and finally encapsulated in a lipid shell with specific recognition capacity by liver cells [139].

**Figure 5:**
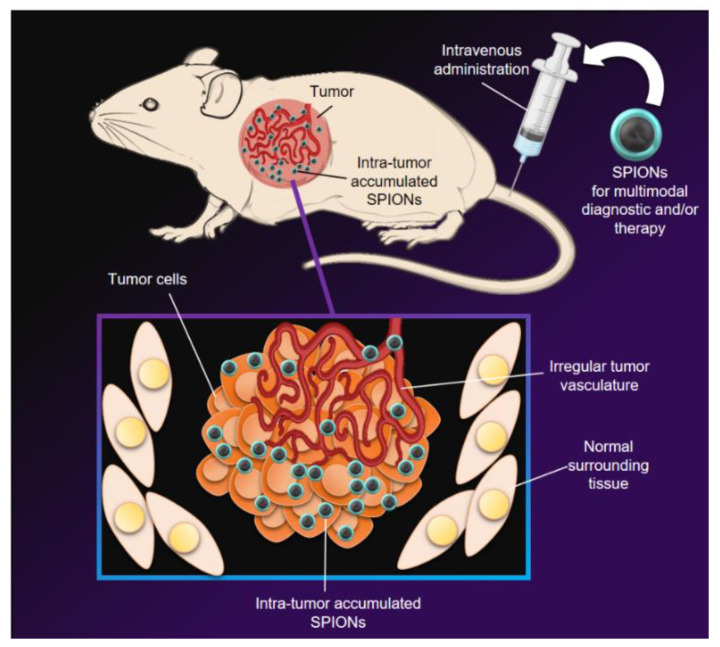
SPIONs intended for multimodal diagnostic and/or therapy are injected via IV. Subsequently, SPIONs are subject to a certain time of blood circulation, and thanks to the discontinuous and irregular vasculature of the tumor, the nanoparticles accumulate in the tumor cells, thus being able to accomplish their objectives. This catchment of nanoparticles in the tumor cells is known as passive targeting and is favored by the enhancing permeability retention effect (EPR). This figure is original for this work.

In summary, the studies of magnetite nanoparticles combined with lipids show a low selectivity for the target organ or tissue in the physiological environment. On the other hand, accumulation in tumor tissues would be due to passive targeting mediated by the ERP effect in tumors through fenestrated vessels (Figure 5). Despite this, a great advantage is the low toxicity of these nanosystem configurations. Furthermore, in the absence of external stimuli such as magnetic fields or radiofrequency, they would be innocuous for non-pathological tissues.

### 4.2. Biological Analysis of Magnetite-Based SPIONs Functionalized with Other Inorganic Materials

According to evidence, it is possible to potentiate hyperthermia therapy by combining magnetic nanoparticles with other inorganic materials. In this regard, Jadhav and colleagues synthesized gadolinium (Gd) doped manganese zinc ferrite magnetic nanoparticles intended for magnetic fluid hyperthermia therapy. They reported that these nanosystems are superparamagnetic and exhibited a high specific loss power (SLP, 146 W/g) that is translated in the potential to rapidly generate heat for future treatments without damaging the surrounding tissue. Initial in vitro studies showed that, in the absence of an external stimulus for heating induction, the nanoparticles were nontoxic for A549 cells derived from human alveolar adenocarcinoma [140]. From the point of view of diagnostic applicability, SPIONs present limitations for MRI studies.

On the other hand, Gd compounds, currently the most used contrast agents, can induce toxicity. A recent study shows the synthesis of a novel multifunctional theranostic system composed of Fe_3_O_4_ nanoparticles coated with Gd ions and decorated with a layer-polycyclodextrin to reduce toxicity. These carriers were also loaded with the anti-tumor drug Curcumin. The unloaded SPIONs were biosecure for normal MCF 10A breast cells or 4T1 cells, which are derived from mouse mammary gland tissue. However, Curcumin-loaded nanoparticles induced deleterious effects more pronounced in tumor cells. Despite this, free Curcumin treatment induced a more significant impact in the reduction of cell viability. This effect was attributed to the lipophilic nature of the drug, which promotes the intake through the phospholipidic cell membrane. These results were considered an advantage since the drug administration through SPIONs could avoid unspecific undesired toxicity. In vivo assessment of the nanoparticles revealed an enhancement of both T1 and T2 contrast in subcutaneous tumors of mice, generated by 4T1 cells implantation in the right flanks. Moreover, the tumor shrinking was registered, and it was highly significant compared to free Curcumin treatment [141].

More studies that implemented Gd with iron oxide nanocomposites have shown that SPIONs combined with gadolinium borate resulted in a pronounced decrease in Gd3 + associated toxicity on tumor cell lines. In this way, it was generated a biocompatible nanoplatform capable of generating dual contrast (T1 + T2) for MRI studies. These formulations were combined with a fluorescent agent (FITC) to facilitate their detection. Folic acid was also added to increase the selectivity for over-expressed folate receptors in MIA-Pa-Ca-2 pancreatic tumor cells, HeLa cervical cancer cells, and A549 cells, thus promoting the specific internalization of nanoparticles in vitro. It was also determined the role of the negative zeta potential, which favors the thorough internalization process. Finally, the Boron included in the nanoformulation provides a potential application for boron Neutron Capture Therapy, based on the nuclear reaction between the B isotope and thermal neutrons [142]. Shabanzadeh-Kouyakhi and colleagues also explored the possibility of generating dual contrast agents with Gd while reducing toxicity. This objective was achieved by combining magnetite SPIONs with Gd and functionalization with dextrose [143].

Gold is another inorganic material capable of interacting with magnetic nanoparticles to increase their suitability and stability for biomedical applications. This is achieved by adding an external Au layer to the iron oxide core [144]. For instance, Fe_3_O_4_/Au MNPs can be used as contrast agents in the dual-modality CT/MRI to detect diseases in the liver. The in vivo imaging of normal mice showed that the nanoparticles provided excellent contrast enhancement for RMI. Moreover, CT studies also demonstrated a good performance of the nanocomposites. For this study, three diseases were used as a model: fatty liver, cirrhotic liver, and hepatocellular carcinoma, and in all cases, the advance of the disease was tracked [145]. Regarding therapy, MNPs coated with Au have been designed to deliver Doxorubicin [144]. Moreover, these nanoparticles could be suitable for two different hyperthermia applicability: magnetically induced hyperthermia ascribed to the iron oxide component and on the other hand photo induced hyperthermia due to the gold presence [146].

According to the overview above we can summarized that the surface functionalization and stabilization of iron oxide nanoparticles is the pivotal step in the broad application and capability of the explored systems.

**Figure 6:**
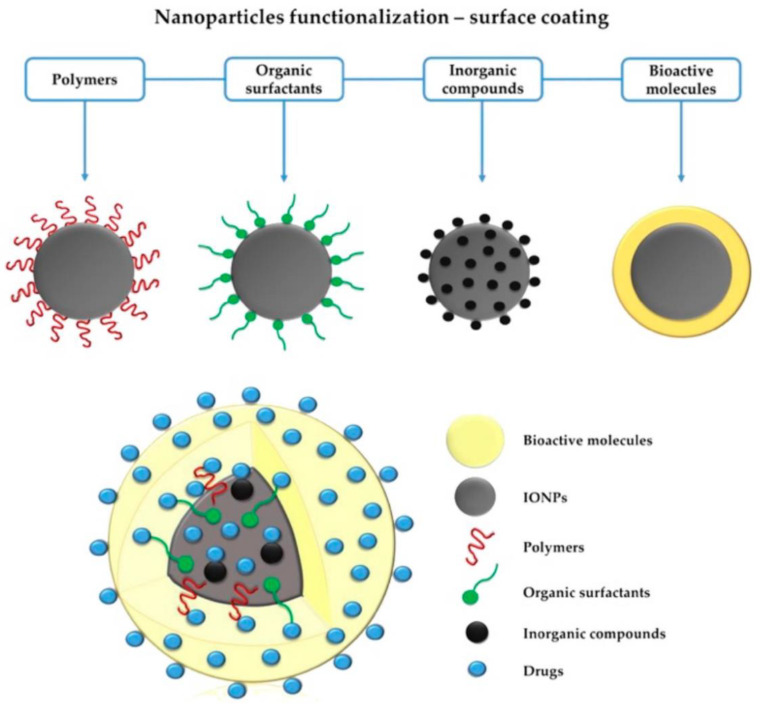
Schematic representation of the modification paths for the surface properties of SPIONs. (Reproduced from ref [147] published by MDPI, 2019).

A schematic overview about the types of the coating corona or ligand (Figure 6, [147]) not only is of a reason for into more biocompatible applications and but as well for the attached to specificity mechanisms towards the biological environmental.

## 5. Concluding Remarks and Future Perspectives

Magnetic nanoparticles for biological applications are seeing a significant increase in research and development in recent years. The potential to control the particle size, shape, and surface functioning has aroused much more interest in their potential application. Magnetic nanoparticles benefit from being utilized in applications such as hyperthermia treatment and being targeted to a specific location within the body to use an alternating magnetic field. MNPs have a large surface area to volume ratio, allowing for high substitution levels, making them suitable for drug delivery. Furthermore, their ability to be incorporated into composites such as magnetic hydrogels or liposomes increases their biocompatibility and potential medical applications.

Although much research has been carried out in animal models on magnetic hyperthermia and drug delivery using MNPs, translating this into suitable treatments for human patients remains a significant challenge. Before MNPs can be widely was using, the effects of their size, shape, substitution, and dosage must be evaluated. Toxicity issues need to be addressed and resolved. Even though initial applications of MNPs in hyperthermia and drug delivery treatments have been successful in the laboratory, extensive in vitro and in vivo trials and also toxicity and long-term, MNP stability tests are required before clinical use.

However, MNPs are synthesized and functionalized almost to order. A large number of proof-of-concept trials have determined their potential as a new species of effective therapeutic agents for many debilitating and life-threatening illnesses. These types of therapy appear to be on the frontlines of becoming much more common in the future.

Critical and constructive investigation is warranted to design and fabricate MNPs for diverse applications in diverse fields in order to overcome challenges. With the advancement of nanotechnology and the development of multidisciplinary approaches, it is essential to consider the formation of regulatory institutions to ensure the safe and effective use of nanotechnology. A common mechanism and connection must be established between institutions and researchers to develop specific standards and platforms for in-vivo clinical trials and pre-clinical studies. MNPs face plenty of challenges if those are to be used in cancer treatment and to combat multi-drug resistance. One of the significant issues is the long-term biocompatibility and toxicity of MNPs. The scientific community should always address such massive challenges and conduct straightforward clinical trials to develop and construct MNPs for a positive outlook.

## Figures and Tables

**Table 1:** Evolution of Z potential for nanosystems prepared with increasing proportions of MNPs, as a function of storage time.

Time/Days	Z Potential (mV)					
	mg MNPs/mL NLCS				NLCs	MNPs
	0.064	0.128	0.32	0.64		
0	−14	−12	−4.5	5.9	−34	45
1	−15.5	−12	−4.8	6.3	−25	51
3	−16	−12	−2.7	7.3	−32.8	50
7	−16	−11	−2.5	7.5	−30.2	50
10	−11.6	−3.4	2.6	7.6	−31.3	52.3
15	−3.9	−1.56	2.7	8.8	−33.3	51.7
